# The Impact of Resveratrol and Melatonin on the Genome and Oxidative Status in Ageing Rats

**DOI:** 10.3390/nu17071187

**Published:** 2025-03-28

**Authors:** Marko Gerić, Lucia Nanić, Vedran Micek, Ivana Novak Jovanović, Goran Gajski, Dubravka Rašić, Tatjana Orct, Marija Ljubojević, Dean Karaica, Jasna Jurasović, Ivana Vrhovac Madunić, Maja Peraica, Ivan Sabolić, Vanessa Moraes de Andrade, Davorka Breljak, Ivica Rubelj

**Affiliations:** 1 Division of Toxicology, Institute for Medical Research and Occupational Health, 10000 Zagreb, Croatia; 2 Laboratory for Molecular and Cellular Biology, Division of Molecular Biology, Ruđer Bošković Institute, 10000 Zagreb, Croatia; 3 Animal Breeding Unit, Institute for Medical Research and Occupational Health, 10000 Zagreb, Croatia; 4 Division of Occupational and Environmental Health, Institute for Medical Research and Occupational Health, 10000 Zagreb, Croatia; 5 Laboratory of Translational Biomedicine, Graduate Program of Health Sciences, University of Southern Santa Catarina–UNESC, Criciúma 88806-000, Brazil; vma@unesc.net

**Keywords:** ageing, resveratrol, melatonin, telomeres, oxidative stress, DNA damage

## Abstract

Background: Given the growing challenges posed by an ageing population, particularly in Western countries, we aimed to investigate the potential geroprotective effects of resveratrol and melatonin in ageing rats. Methods: The animals were treated with these two compounds starting at 3 months of age and continuing until 1 year or 2 years of age. Using a multibiomarker approach, we assessed DNA damage, telomere length, and the oxidative status in their urine, liver, and kidneys. Results: Despite employing this experimental approach, our results did not provide conclusive evidence of geroprotective effects across the evaluated organs. However, we observed sex-dependent differences in response to treatment. Conclusions: Given the high potency of these two compounds, further research is warranted to explore their incorporation into daily routines as a strategy to mitigate ageing-related effects.

## 1. Introduction

Ageing is one of the most pronounced demographic features of the modern human population. According to WHO data, the number of people older than 60 will double by 2050. Moreover, the proportion of people older than 80 is growing at an even faster rate. As life expectancy increases, it often reflects improved health status. However, as suggested by the WHO’s Healthy Ageing Concept, it is important to maintain and improve intrinsic capacity, functional ability, and supportive environments for an ageing population [1,2].

At the cellular and molecular levels, ageing in mammals is primarily driven by the accumulation of senescent cells in tissues and organs, among other contributing factors [3]. Cellular senescence can be categorised into two distinct types. The first is replicative senescence, caused by the progressive shortening of telomeres during successive cell divisions. The second is stress-induced premature senescence, which can result from the interplay of various factors, including DNA damage, oxidative stress, or oncogene activation [4,5,6]. Additionally, stress can cause abrupt telomere shortening, triggering cells to enter senescence in a sudden and stochastic manner [7,8]. Preventing telomere shortening, the region that protects the ends of linear chromosomes is essential to avoid chromosomal rearrangements and preserve the stability of the genome [9].

People have been trying to find the so-called Fountain of Youth for thousands of years. In a quote nearly as old, Hippocrates emphasised the importance of food as medicine and a key in promoting good health and longevity. During the last couple decades, several compounds have gained attention from both the public and scientists due to their geroprotective properties. The consumption of cruciferous vegetables, green tea, turmeric, tomatoes, onion, leek, and garlic are often encouraged to enrich the diet with bioactive compounds such as sulforaphane, phenethyl isothiocyanate, epigallocatechin-3-gallate, curcumin, sulphur and selenium-rich compounds, and lycopene, all of which have demonstrated significant chemopreventive potential [10].

Resveratrol (RSV), a plant-derived stilbenoid, also belongs to the class of the above-mentioned compounds. This potent molecule is found mostly in grapes, peanuts, and berries, while its presence in a more favourable, bioavailable form is predominantly found in red wine, as ethanol enhances its extraction and solubility [11]. Studies have shown that RSV postpones cellular senescence, reduces the rate of telomere shortening, and enhances telomerase activity. Furthermore, RSV modulates neuroactive, calcium-dependent, and cancer-related signalling pathways, as well as nitrogen metabolism, contributing to various health benefits in humans and lifespan extension in model organisms such as *Saccharomyces cerevisiae*, *Caenorhabditis elegans*, and *Drosophila melanogaster* [12,13,14,15,16,17]. However, despite the extensive body of evidence, the overall health benefits of RSV supplementation remain inconclusive.

Melatonin (MEL), although present in some edible plants [18], is predominantly produced by the pineal gland; however, its synthesis in smaller amounts is recorded in various mammalian organs. This indolamine is usually related to the regulation of circadian rhythm. Additionally, it is a highly evolutionary conserved molecule, suggesting a wide range of protective properties for the host organism. By interacting with both membrane receptors and cytosolic targets, MEL plays a crucial role in regulating the expression of antioxidant pathways, directly scavenging free radicals, and modulating anti-inflammatory responses [19,20]. The dual role of MEL has been observed in cancerous or damaged cells, where it can promote a pro-oxidant state, often activating apoptosis pathways. Nevertheless, many studies and its long-term use in humans have so far failed to identify toxic properties. On the contrary, MEL has demonstrated benefits in cardio-protection, metabolic syndrome management, brain health, and ageing [21].

Studying ageing and the potential ageing-ameliorating compounds in humans is extremely challenging and expensive, often encountering methodological and statistical limitations [22]. Therefore, we aimed to investigate the potential beneficial properties of RSV and MEL supplementation in ageing Wistar rats [23,24,25]. The effects of these compounds were assessed in terms of DNA damage and telomere dynamics, recognised as hallmarks of ageing [26], as well as oxidative status in the liver and kidneys during chronic treatment in one- and two-year-old rats.

## 2. Results

The experimental design to study ageing, using rats as a model organism, was implemented in our animal breeding unit. Starting at three months of age, experimental animals of both sexes were treated with RSV (10 mg/L) or MEL (10 mg/L), dissolved in 0.1% ethanol, for either 9 months or 21 months, until they reached 1 year or 2 years of age, respectively. Based on our previously published results using the same ageing model, the average MEL consumption was 378 µg/kg body mass (b.m.)/day, while the average RSV consumption was 300 µg/kg b.m./day [24].

**Figure 1:**
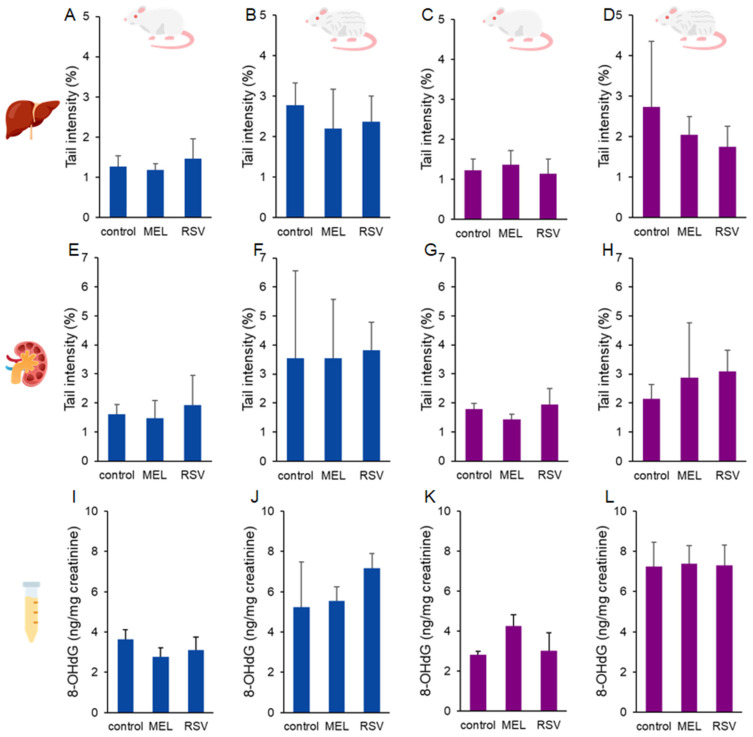
DNA damage results. DNA strand breaks in the liver (**A**–**D**) and kidney (**E**–**H**), along with urinary levels of 8-oxo-2′-deoxyguanosine (8-OHdG) (**I**–**L**), measured in ageing rats (1-year-old and 2-year-old, both sexes) treated with melatonin (MEL) or resveratrol (RSV). Blue bars represent male rats, while purple bars represent female rats. The first and third columns correspond to 1-year-old animals, whereas the second and fourth correspond to 2-year-old animals. No statistically significant differences were observed between the treated and control groups.

The effects of these two compounds on ageing rats were not detected in terms of DNA damage or oxidative changes to guanine (Figure 1). The percentage of DNA damage in liver and kidney cells, as measured by the comet assay, ranged from 1.18 to 3.82%, with no significant differences observed between the experimental groups. Similarly, urinary levels of 8-oxo-2′-deoxyguanosine (8-OHdG) ranged from 2.76 to 7.38 ng/mg creatinine, with no significant differences among the groups.

**Figure 2:**
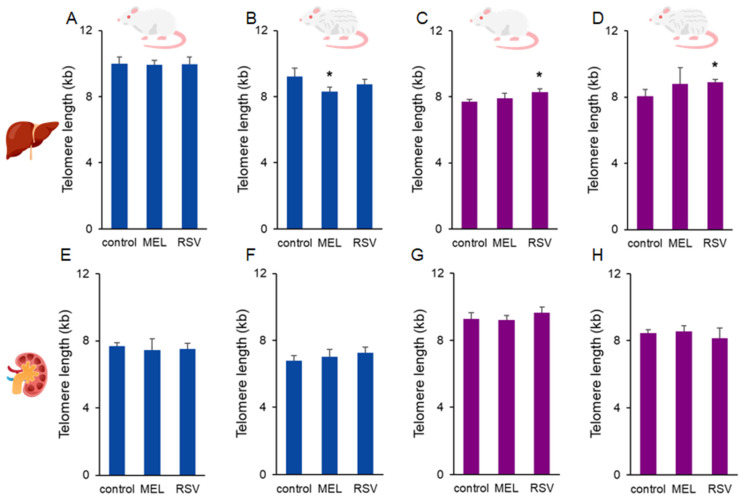
Average telomere length in the liver (**A**–**D**) and kidney (**E**–**H**) of ageing rats (1-year-old and 2-year-old, both sexes) treated with melatonin (MEL) or resveratrol (RSV). Blue bars represent male rats, while purple bars represent female rats. The first and third columns correspond to 1-year-old animals, whereas the second and fourth correspond to 2-year-old animals. * indicates significantly different (*p* < 0.05) compared to the control group.

Long-term treatment with RSV positively affected telomere length in the liver of female rats. The average telomere length in 1- and 2-year-old untreated animals was 7.71 ± 0.14 and 8.06 ± 0.42 kb, respectively, whereas treatment maintained their telomere length at 8.29 ± 0.17 and 8.92 ± 0.14 kb, respectively (*p* < 0.05). No significant effects of MEL were observed in the liver in female rats. Conversely, in 2-year-old male rats, MEL decreased telomere length in the liver compared to the corresponding control group (9.22 ± 0.51 vs. 8.32 ± 0.25 kb, *p* < 0.05). The effect of RSV on telomeres in the liver of male rats, as well as the effects of both compounds on telomeres in both sexes, was not observed in kidney samples (Figure 2).

**Figure 3:**
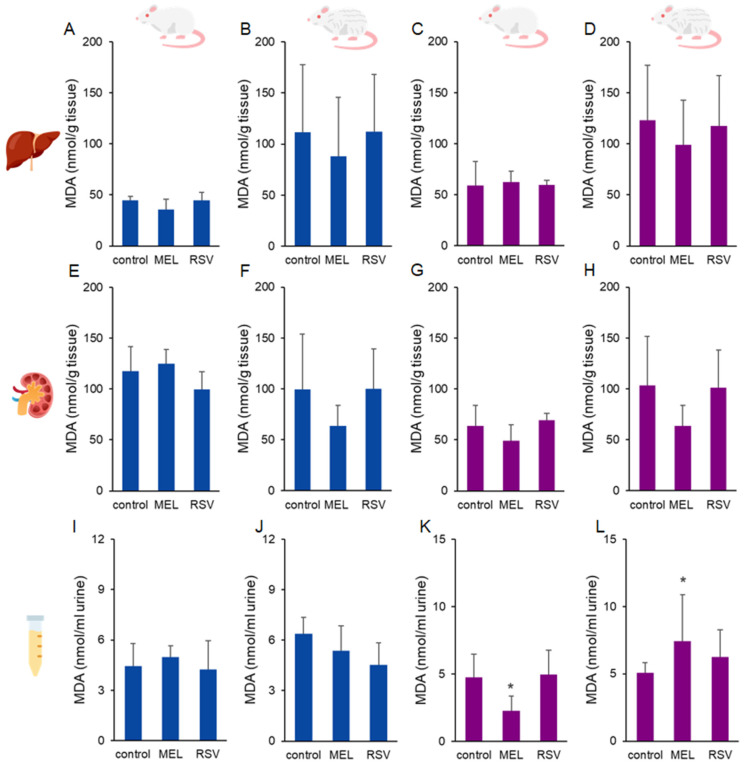
Malondialdehyde (MDA) concentrations in the liver (**A**–**D**), kidney (**E**–**H**), and urine (**I**–**L**) of ageing rats (1-year-old and 2-year-old, both sexes) treated with melatonin (MEL) or resveratrol (RSV). Blue bars represent male rats, while purple bars represent female rats. The first and third columns correspond to 1-year-old animals, whereas the second and fourth correspond to 2-year-old animals. * indicates significantly different (*p* < 0.05) compared to the control group.

As a marker of oxidative stress, malondialdehyde (MDA) levels were measured in the liver and kidney of rats (Figure 3), with no significant differences observed between MEL- or RSV-treated animals. Tissue MDA levels ranged from 36.18 ± 2.49 to 123.08 ± 54.12 nmol/g tissue. However, differences were observed in urinary MDA levels in female rats treated with MEL. After 9 months of treatment, urinary MDA levels were lower (2.26 ± 1.09 nmol/mL urine) compared to the control group (4.75 ± 1.73 nmol/mL urine). This trend, however, reversed after the longer treatment period of 21 months, where MEL-treated rats exhibited higher urinary MDA levels (7.43 ± 3.44 nmol/mL urine) compared to the control group (5.06 ± 0.76 nmol/mL urine).

**Figure 4:**
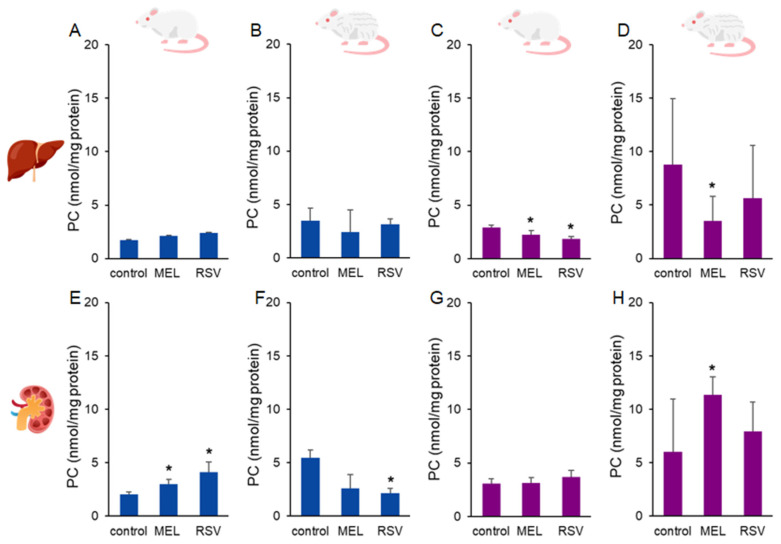
Protein carbonyl (PC) concentrations in the liver (**A**–**D**) and kidney (**E**–**H**) of ageing rats (1-year-old and 2-year-old, both sexes) treated with melatonin (MEL) or resveratrol (RSV). Blue bars represent male rats, while purple bars represent female rats. The first and third columns correspond to 1-year-old animals, whereas the second and fourth correspond to 2-year-old animals. * indicates significantly different (*p* < 0.05) compared to the control group.

Another biomarker of oxidative stress, protein carbonyls (PC) was assessed in the liver and kidney of treated ageing rats (Figure 4). No significant effects of the treatments were observed in the liver of male rats. In contrast, female rats exhibited lower liver PC levels (*p* < 0.05) after 9 months of treatment with MEL (2.20 ± 0.39 nmol/mg protein) or RSV (1.82 ± 0.22 nmol/mg protein) compared to the control group (2.89 ± 0.21 nmol/mg protein). After the longer treatment period, the reduction in the liver PC levels persisted in MEL-treated female rats (3.49 ± 2.29 nmol/mg protein) compared to the control group (8.77 ± 6.16 nmol/mg protein). In 1-year-old male rats, kidney PC levels were higher (*p* < 0.05) in both MEL-treated (3.01 ± 0.42 nmol/mg protein) and RSV-treated (4.09 ± 0.98 nmol/mg protein) groups compared to the control group (2.01 ± 0.24 nmol/mg protein). However, in 2-year-old male rats, the MEL-treated group (2.62 ± 1.28 nmol/mg protein) did not differ from the control group (5.46 ± 0.74 nmol/mg protein), while the RSV-treated group exhibited lower kidney PC levels (2.17 ± 0.40 nmol/mg protein, *p* < 0.05). In female rats, kidney PC levels were not significantly affected by MEL or RSV treatments in 1-year-old animals. However, in 2-year-old female rats, the MEL-treated group exhibited higher kidney PC levels (11.35 ± 1.67 nmol/g tissue, *p* < 0.05) compared to the control group (6.02 ± 4.94 nmol/mg protein). RSV treatment did not significantly affect kidney PC levels in this group.

**Figure 5:**
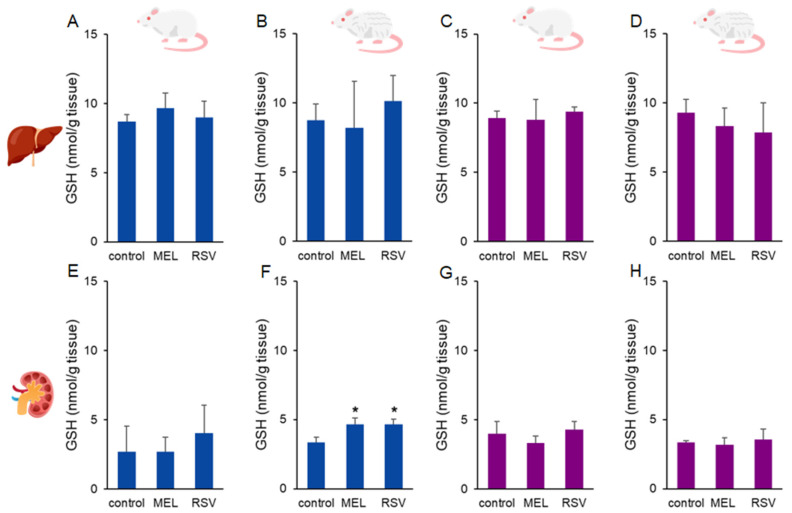
Glutathione (GSH) concentrations in the liver (**A**–**D**) and kidney (**E**–**H**) of ageing rats (1-year-old and 2-year-old, both sexes) treated with melatonin (MEL) or resveratrol (RSV). Blue bars represent male rats, while purple bars represent female rats. The first and third columns correspond to 1-year-old animals, whereas the second and fourth correspond to 2-year-old animals. * indicates significantly different (*p* < 0.05) compared to the control group.

To assess oxidative stress defence, we evaluated the impact of MEL and RSV treatment on glutathione (GSH) levels in rat tissues. Higher GSH levels were observed in the kidneys of 2-year-old male rats treated with both MEL (4.65 ± 0.45 nmol/g tissue) and RSV (4.63 ± 0.38 nmol/g tissue) compared to the control group (3.36 ± 0.37 nmol/g tissue). No significant differences were detected in liver GSH levels or kidney GSH levels of female rats after long-term treatment with both compounds (Figure 5).

**Figure 6:**
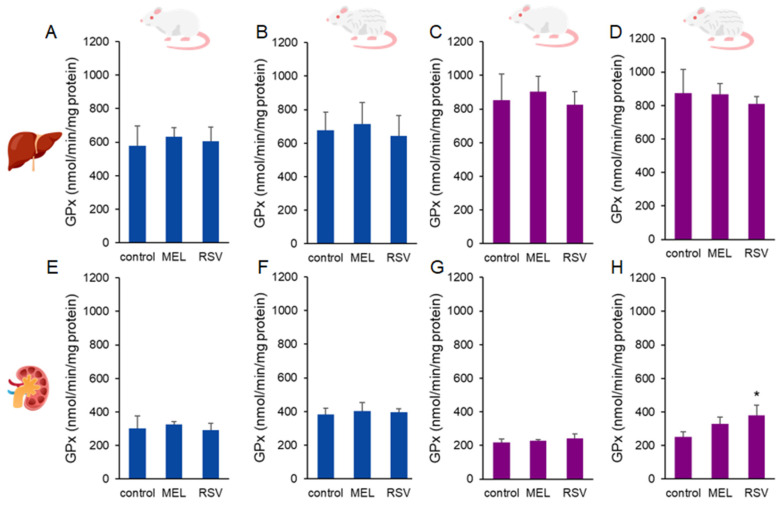
Glutathione peroxidase (GPx) activity in the liver (**A**–**D**) and kidney (**E**–**H**) of ageing rats (1-year-old and 2-year-old, both sexes) treated with melatonin (MEL) or resveratrol (RSV). Blue bars represent male rats, while purple bars represent female rats. The first and third columns correspond to 1-year-old animals, whereas the second and fourth correspond to 2-year-old animals. * indicates significantly different (*p* < 0.05) compared to the control group.

**Figure 7:**
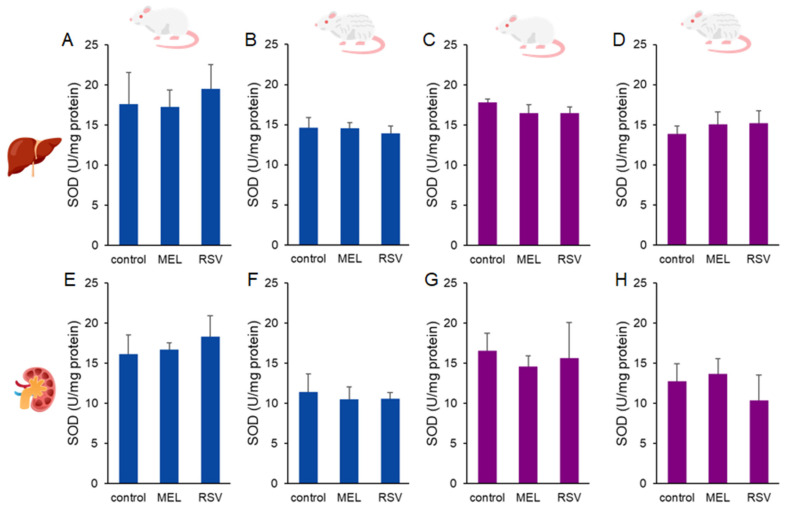
Superoxide dismutase (SOD) activity in the liver (**A**–**D**) and kidney (**E**–**H**) of ageing rats (1-year-old and 2-year-old, both sexes) treated with melatonin (MEL) or resveratrol (RSV). Blue bars represent male rats, while purple bars represent female rats. The first and third columns correspond to 1-year-old animals, whereas the second and fourth correspond to 2-year-old animals. No statistically significant differences were observed between the treated and control groups.

Additionally, we evaluated two key enzymatic defences against oxidative stress: glutathione peroxidase (GPx) and superoxide dismutase (SOD). Increased GPx activity was observed in the kidneys of 2-year-old female rats treated with RSV (377.89 ± 64.00 nmol/min/mg protein) compared to the control group (251.37 ± 30.28 nmol/g tissue). No significant differences were detected in liver GPx activity or in kidney GPx activity of males after long-term treatment with either compound (Figure 6). Regarding SOD activity, no significant differences were observed across sexes or treatment groups in ageing rats (Figure 7).

## 3. Discussion

In the present study, we investigated the impact of long-term treatment with melatonin (MEL) and resveratrol (RSV) on the organs of ageing rats. Using a multi-biomarker approach, we observed distinct sex-dependent responses to the treatment, emphasising the importance of including animals of both sexes in in vivo studies [27,28]. Previously, we reported the effects of MEL and RSV on blood biomarkers and, contrary to the current findings, generally did not observe significant differences in DNA damage and oxidative stress biomarkers following the treatment [24]. These discrepancies may be attributed to various sex-specific differences reported in the literature [29] and organ-specific physiology, which we will further elaborate upon in the following paragraphs.

Regarding MEL treatment, we observed sex-dependent differences primarily in oxidative stress biomarkers. Notably, 1-year-old female rats treated with MEL exhibited a specific physiological change, consuming 2–3 times more liquid compared to other groups, which resulted in higher MEL exposure (up to 1200 µg/kg b.m./day). While MEL targets multiple pathways in the organism, leading to diverse health outcomes, it likely acted as an antioxidant in this case, reducing oxidative damage to lipids (MDA) and proteins (PC) as detected in urine and kidney samples. In contrast, 2-year-old female rats consumed MEL at levels similar to other groups, resulting in relatively lower exposure compared to the 1-year-old group. Additionally, although the main MEL receptors (MT_1_ and MT_2_) tend to downregulate their mRNA and protein levels with age, an interesting phenomenon of endogenous extrapineal MEL production has been observed in several tissues [30]. In aged rats, the liver tends to decrease the levels of MEL-producing enzymes, leading to reduced MEL concentrations in liver tissues. On the other hand, the ageing rat kidney exhibits an opposite response, with MEL-producing enzymes and MEL tissue concentrations increasing with age [31]. Moreover, MEL is considered a conditional pro-oxidant, since in some in vitro studies and at high cellular concentrations (µM to mM range), it has been shown to stimulate reactive oxygen species production processes and induce growth arrest [21]. These mechanisms might contribute to the observed increase in PCs and MDA levels in the kidney and urine of 2-year-old female rats (Figure 3 and Figure 4), respectively. Conversely, liver PC levels remained lower in the MEL-treated group compared to the untreated control group. No significant differences were observed among the groups in terms of DNA damage, oxidative DNA damage, and telomere length

The oxidative status in male MEL-treated rats exhibited some differences compared to females. In 1-year-old male rats, kidney PC levels were higher than those in the control group. It is plausible that these changes stimulated an increase in GSH levels to counteract ROS generation, as elevated GSH levels were still observed in 2-year-old rats. This non-enzymatic oxidative defence mechanism likely contributed to maintaining PC levels in the kidneys similar to those of untreated animals. A similar pattern was observed in liver telomere length, which was shorter in 1-year-old rats but showed no differences in 2-year-olds. The liver has been identified as a major site for MEL metabolism, while most MEL excretion occurs via the kidneys in rats [32].

When compared to other studies, MEL demonstrated the ability to alleviate ageing-induced DNA damage in the blood, liver, and kidneys, while also extending the survival of supplemented mice [33,34]. Additionally, MEL has been shown to promote reproductive health in female mice by reducing oxidative damage [35]. On the other hand, some studies did not report significant beneficial properties in heart tissue, particularly in terms of MDA and GSH levels [36]. In the cases of protection against polycyclic aromatic hydrocarbons, arsenic exposure, or induced maple syrup urine disease, ageing, or pneumonia, MEL has been shown to reduce DNA strand breaks, decrease telomerase reverse transcriptase activity, and improve oxidative stress defence parameters using in vitro cell models as well as in vivo rodent models. However, these effects were often strongly tissue-specific [37,38,39,40,41].

Melatonin has been extensively studied for over three decades across molecular, cellular, animal, and human models for its potential role in various medical conditions. While the majority of publications support the beneficial properties of MEL, there are relatively few reports demonstrating health benefits that can be directly translated into clinical practice [42]. Some of the evidence accumulated over time may be influenced by publication bias, where positive findings are more likely to be published, as well as studies that replicate previously reported results [43,44]. In our study, we occasionally observed the induction of oxidative damage to lipids and proteins after long-term treatment with MEL. This finding is noteworthy, as relatively few studies have reported potentially negative effects of MEL. When considering translational applications to humans, it is essential to establish dose-response curves and EC_50_ values for different effects. Such data would enhance the interpretation of results and clarify the potential benefits of MEL supplementation.

In the RSV-treated groups, sex differences were also observed. In female rats, the RSV treatment preserved telomere length in both age groups, suggesting potential beneficial effects. Maintaining longer telomeres is critical for preventing cellular senescence and the ageing phenotype of certain tissues or organisms. In cells with intact signalling pathways and functional checkpoints, critical telomere shortening triggers cellular senescence or apoptosis. However, if these checkpoints fail, the cell may escape normal ageing endpoints, leading to a state associated with genomic rearrangements, DNA damage, oxidative stress, and, subsequently, carcinogenesis [45,46,47,48]. No significant changes in oxidative status or genome integrity were observed in either treatment group.

Similarly to MEL-treated male rats, 1-year-old RSV-treated male rats also exhibited higher kidney PC levels. However, in 2-year-olds, we observed increased GSH levels and a reduction in PC levels compared to the control group. Despite the ongoing interest in RSV’s beneficial properties, there is still insufficient evidence to support its efficacy in the treatment of any specific disease. Therefore, further studies are necessary to fully elucidate both the mechanisms and effects of RSV supplementation. Our results in the female liver are in accordance with most published results, supporting RSV’s geroprotective properties [10,12]. One of the major challenges with RSV lies in its dosage and bioavailability. Usually, only about 1% of the orally administered dose is detectable in the bloodstream due to its extensive high metabolism in the liver. This biotransformation results in the formation of numerous metabolites, some of which may exhibit even greater biological activity [11]. This explanation could be relevant to the improved telomere status observed in female rats that consumed higher doses of RSV. On the other hand, after years of emphasising RSV’s benefits, several studies have reported that its mode of action may be hormetic in certain conditions, including specific enzyme levels, pH levels, substrate availability, exposure duration, and concentrations, where RSV may act as a pro-oxidant. Additionally, animal studies have highlighted the dual redox behaviour of RSV depending on its consumption during the day or night cycle [49]. A similar scenario could potentially explain the observations in the kidneys of male rats.

When compared to other studies, RSV influenced the liver oxidative status of 2-year-old rats acutely exposed to heat stress. As for the chronically induced conditions, RSV had an impact on liver and vascular telomere length in 1-year-old rats and 36-week-old rats exposed to arsenic [17,50,51]. RSV treatment also mitigated the plasma oxidative status in copper-deficient rats and glyoxal-induced oxidation modifications in rat cells [52,53]. In contrast, mycotoxin-exposed rats exhibited variable responses in blood oxidative status after RSV treatment [54,55,56]. Additionally, no changes were observed in the heart oxidative status of 16-month-old diabetic rats following a 4-week RSV intervention [36].

According to current bibliometric data on the health effects of RSV, over 1600 papers have been published annually over the past decade, highlighting its significant potential for clinical application settings. Regarding the human effects of RSV treatment, the number of registered trials peaked in 2018 and has since declined. However, approximately 67% of these studies reported positive outcomes, while 26% noted side effects comparable to those observed in non-exposed groups. Moreover, about 33% of the studies did not report any side effects [57]. Despite the promising findings, several challenges in RSV research persist, including its bioavailability, solubility, and minimising adverse effects, particularly at higher concentrations. These limitations emphasise the need for continued research in this field, including the exploration of nanotechnology-based strategies for optimising RSV as a therapeutic agent [49,58,59,60].

The results of this study should be interpreted within the context of several limitations. We used relatively small animal groups, which was feasible considering the 3Rs principle and the financial constraints of long-term experiments. Consequently, some interindividual variability may have obscured certain expected outcomes. The administration of MEL and RSV was conducted via ad libitum water intake using only one concentration solution per compound, which allowed only an approximation of the daily intake of the tested compounds. However, this method minimised the potential stress that could have been induced by repeated oral administration.

## 4. Conclusions

Based on our findings, neither MEL nor RSV, at the concentrations used and under the current study design, demonstrated consistent beneficial effects in different organs of both sexes in ageing rats. Drawing a parallel to the concept of the Holy Grail, often depicted as a miraculous artefact with healing powers or a source of eternal youth, our results suggest that instead of relying on a single molecule to preserve health, a more holistic approach may be necessary. This includes adopting quality lifestyle habits, maintaining a balanced diet, and placing greater emphasis on disease prevention as the most effective strategy to address the complexity of longevity [61]. Since the response to MEL or RSV treatment was also strongly sex-related, we might speculate on the impact of hormonal-dependent differences as seen in the other physiological phenomena [62,63,64]. Nevertheless, the potential of these two biomolecules should not be underestimated. Further research is warranted to identify the exact mechanisms of sex differences and optimal methods for integrating MEL and RSV into daily routines to promote health and well-being.

## 5. Materials and Methods

### 5.1. Animals

Wistar rats (outbred strain CRL: WI(Han)) were bred and maintained at the breeding colony of the Institute for Medical Research and Occupational Health (IMROH, Zagreb, Croatia), according to the Directive 2010/63/EU. The housing conditions included standardised environmental parameters: room temperature (20–24 °C), humidity (40–60%), and a 12 h light/dark cycle. Animals were provided with ad libitum access to tap water and certified pelleted food (4RF21, Mucedola, Settimo Milanese, Italy). When the animals reached 3 months of age, they were included in the experimental procedures as described below. All studies were performed in compliance with the ARRIVE guidelines and conducted in accordance with the Guide for the Care and Use of Laboratory Animals of the National Institute of Health following the high standards of the “3Rs” principle. The experimental protocols were approved by the Institutional Ethics Committee (100-21/14-7) and the Croatian Ministry of Agriculture (525-10/0255-15-4).

### 5.2. Animal Treatment and Sample Collection

**Figure 8:**
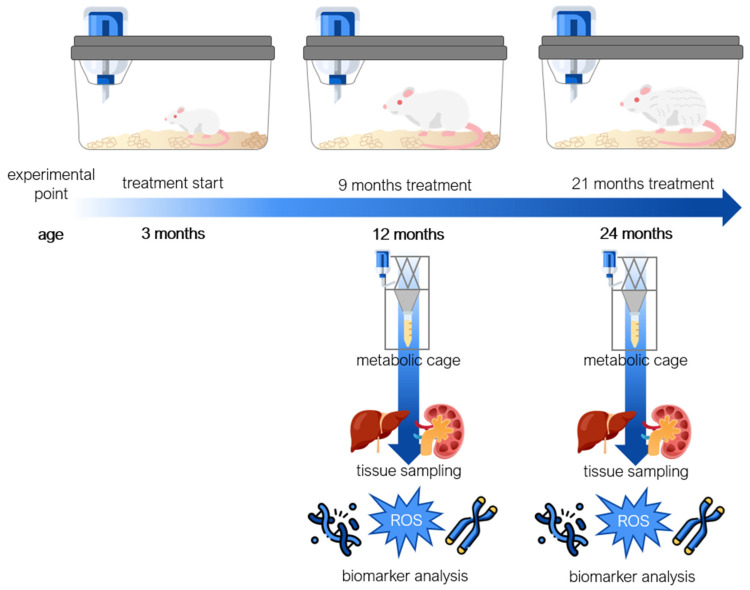
A study and sampling design. Starting at 3 months of age, rats were provided with ad libitum access to either a vehicle (0.1% *v*/*v* ethanol in tap water), melatonin (MEL; 10 mg/L vehicle), or resveratrol (RSV; 10 mg/L vehicle), continuing until they reached 12 or 24 months of age. Prior to sacrifice, the animals were weighed and housed in metabolic cages to assess liquid consumption and collect urine samples. Subsequently, liver, kidney, and urine samples were collected for the analyses of baseline DNA damage, telomere length, and oxidative stress biomarkers.

**Figure 9:**
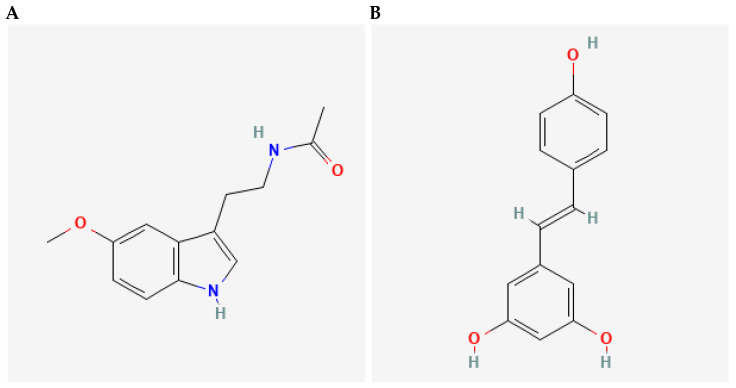
Chemical structures of melatonin ((**A**) IUPAC name *N*-[2-(5-methoxy-1*H*-indol-3-yl)ethyl]acetamide, CAS number 73-31-4) and resveratrol ((**B**) IUPAC name 5-[(*E*)-2-(4-hydroxyphenyl)ethenyl]benzene-1,3-diol, CAS number 501-36-0). Adapted from reference [65].

An overview of study design is shown in Figure 8. Male and female rats, aged 3 months, were randomly assigned to groups of 4 animals per cage (males and females housed separately), and each experimental point consisted of 4 animals (N = 4, 48 animals in total). Sample size was determined based on our previous study [24] and respecting the 3Rs principle. We did not exclude any animals, and there were no spontaneous deaths recorded due to an ageing model, while the criterion to be included in the study was body mass within ±20% of a group average. Under standardised environmental conditions, as described earlier, the animals had ad libitum access to pelleted food and either vehicle control (0.1% ethanol in tap water), RSV (10 mg/L vehicle), or MEL (10 mg/L vehicle) for 9 or 21 months. This experimental setup aimed to investigate the long-term effect of the studied compounds on ageing. MEL (≥98% pure powder, Sigma-Aldrich, Burlington, MA, USA) and RSV (≥99% pure powder; Xi’an Lyphar Biotech Co., Xi’an, China) (Figure 9) were initially dissolved in absolute ethanol at a concentration of 10 mg/mL, followed by a 1000-fold dilution in tap water to obtain drinking solutions containing 10 mg/L of MEL or RSV. To minimise light-induced degradation, both solutions were prepared under dim light and transferred to bottles wrapped in black foil for protection. At the ages of 1 or 2 years, after 9- or 21-month treatments, respectively, all animals were sacrificed, and liver and kidney samples were collected. Samples were stored at −80 °C for subsequent analysis. Urine sampling was conducted as described earlier and in our previous studies [24,29]. Briefly, two days prior to sacrifice, rats were individually placed in the metabolic cages with free access to water but without food. Urine was collected over a 24 h period, then centrifuged at 2500× *g* for 15 min to isolate supernatants, which were then stored at −20 °C for further use. All of the studied groups followed the same handling procedures to minimise the effect of confounders and reduce pain or distress. The study group allocation was blind to the team members, until the data analyses, besides the person who was responsible for the laboratory animals’ housing and handling.

### 5.3. Telomere Detection and Length Analysis

Total DNA from liver and kidney samples was isolated using a DNeasy Blood & Tissue Kit (Qiagen, Hilden, Germany). Genomic DNA was quantified using a nanophotometer (Implen GmbH, München, Germany). Aliquots of genomic DNA (2 μg) were digested overnight at 37 °C with restriction enzymes RsaI/HinfI (NEB, Ipswich, MA, USA) (5 U/μg DNA each), resulting in cleaved genomic DNA fragments containing intact telomere regions (Terminal Restriction Fragment, TRF). Post-digestion, DNA was precipitated, and equal amounts (1.2 μg) of DNA were loaded onto a 1% agarose gel. The samples were separated via pulsed-field gel electrophoresis (Chef-DR III; Bio-Rad, Hercules, CA, USA) at 6 V/cm with a switching time of 1–30 s for 7 h at 14 °C. DNA Molecular Weight Marker II, DIG-labelled (Sigma-Aldrich) was run in each gel. After electrophoresis, gels were stained with ethidium bromide to confirm complete digestion. DNA was then transferred onto positively charged nylon membranes (Roche, Basel, Switzerland) via overnight capillary transfer at room temperature using 20X SSC buffer. Membranes were hybridised overnight at 68 °C with a digoxigenin-labelled telomere-specific probe. Telomere digoxigenin-labelled probe was prepared by non-template PCR using primers specific for the telomere sequence F (CCCTAA)4 and R (TTAGGG)4 (95 °C/1 min, 55 °C/1 min 40 s, 72 °C/1 min 50 s; 30 cycles, 72 °C/7 min). After hybridisation, membranes were incubated with an anti-digoxigenin AP-conjugate (Roche) and with CDP-Star (Roche) for chemiluminescent detection of telomere repeats. Signals were visualised using X-ray film (Agfa, Mortsel, Belgium), and the intensity of the telomere signals was quantified by densitometry with Image Master VDS Software version 2.0 (Amersham Biosciences, Amersham, UK). Obtained raw data were used to calculate the average telomere length in each sample using the formula Σ(ODi − background)/Σ(ODi − background/Li), where ODi is the chemiluminescent signal, and Li is the length of the TRF at position i [66].

### 5.4. Alkaline Comet Assay Procedure

The comet assay is a sensitive method for detecting DNA damage in individual cells under various toxic and oxidative stress-related conditions in humans and experimental animals [67,68,69]. The alkaline version of the comet assay was performed on liver and kidney cell suspension according to the protocol by Collins et al. [70] following the MIRCA guidelines [71]. Tissue samples were gently homogenised in a prechilled PBS buffer and embedded in 0.5% low-melting-point agarose (Sigma, Burlington, MA, USA), which was carefully layered on top of a pre-solidified 0.6% normal-melting-point agarose (Sigma) base. The slides were incubated overnight at 4 °C in a lysis solution containing 10% DMSO, 2.5 M NaCl, 1% sodium *N*-lauroyl sacosinate, 100 mM (all from Kemika, Zagreb, Croatia), Na_2_EDTA, 10 mM Tris, and 1% Triton X-100 (all from Sigma) adjusted to pH 10. Following lysis, the slides were transferred to an electrophoresis solution (300 mM NaOH, 1 mM Na_2_EDTA, pH 13) for 20 min at 4 °C to allow DNA unwinding. Electrophoresis was then conducted for 20 min at 1 V/cm. After electrophoresis, the slides were washed three times using Tris-HCl buffer, stained with ethidium bromide (10 μg/mL, Sigma), and stored at 4 °C until analysis, which was performed within 2 h. A total of 100 randomly captured nuclei per animal on coded slides were analysed using the Comet Assay II image analysis software (Perceptive Instruments Ltd., Haverhill, Suffolk, UK). As the comet assay descriptor, we chose a tail intensity (% tail DNA) that corresponded to the percentage of DNA migrated in the comet’s tail. A mean of 100 scores per animal was used, and a mean of all animals represented the final score.

### 5.5. Urinary 8-OHdG

Prior to analysis, frozen urine samples were thawed overnight at 4 °C and centrifuged at 2000× *g* for 10 min at 4 °C. The supernatants were then diluted 1:2 with phosphate-buffered saline (PBS, pH 7.4). The level of urinary 8-OHdG (8-hydroxy-2′-deoxyguanosine) was measured using an ELISA kit (Highly sensitive 8-OHdG Check, ELISA kit, JaICA, Tokyo, Japan). Calibration, curve fitting, and data analysis were performed following the manufacturer’s instructions. Creatinine concentration was measured using the spectrophotometric Jaffe’s method [72]. Results are expressed as ng 8-OHdG per mg of creatinine.

### 5.6. Glutathione Analysis

Glutathione (GSH) levels were analysed using Ellman’s method [73]. Kidney and liver homogenates (10%) were prepared in 0.3 M sodium phosphate buffer (Na-P buffer). To 300 µL of the supernatant, 100 µL of 5% trichloracetic acid (TCA) was added. The homogenates were then mixed and centrifuged at 3500 rpm for 10 min. For the assay, 100 µL of either blank solution (H_2_O), standards, or samples (kidney and liver supernatants and plasma) were mixed with 850 μL phosphate buffer and 50 μL 5,5′-ditiobis-(2-nitrobenzoic acid) (DTNB, Sigma). The absorbance of blank solution, standards, and samples was measured spectrophotometrically at 412 nm using a Cecil 9000 spectrophotometer (Cambridge, UK). GSH concentration in the samples was calculated from a standard calibration curve expressed as nmol per gram of tissue.

### 5.7. Malondialdehyde Analysis

The method for MDA analysis in the kidney and liver was based on Drury et al. [74]. Kidney and liver homogenates (10%) were prepared in 0.3 M Na-P buffer solution. For each sample (50 μL of kidney and liver homogenates or plasma) or standard (2.5 μM 1,1,3,3-tetraethoxypropane), the following reagents were added: 5 μL butylated hydroxytoluene (BHT, Sigma) (0.2%, *w*/*v*), 750 µL of phosphoric acid (1%, *v*/*v*), 250 µL of 2-thiobarbituric acid (TBA, Sigma) (0.6%, *w*/*v*), and 445 µL water. The samples were thoroughly mixed and incubated in a boiling water bath for 30 min. The high-performance liquid chromatography (HPLC) system included a degasser, an isocratic pump, a column oven, and a UV detector (Shimadzu Corporation, Kyoto, Japan). The guard and analytical columns were reverse-phase C 18 (Zorbax Eclipse Plus, Agilent, Santa Clara, CA, USA) with 5 μm particles (4.6 × 12.5 and 4.6 × 100 mm, respectively). The mobile phase consisted of 50 mM KH_2_PO_4_ and methanol (60:40, *v*/*v*, pH 6.8). The analysis conditions were as follows: injection volume, 20 μm; flow rate, 1 mL min^−1^; absorbance at UV detector, 532 nm; and temperature in the column oven, 32 °C. Under these conditions, the retention time of MDA was approximately 2.5 min. The MDA concentration was calculated using calibration curves generated with known standards and analysed by HPLC.

### 5.8. Protein Carbonyls

Protein oxidation in kidney and liver tissue homogenates (10% in 0.3 M Na-P buffer solution) was assessed following the method described by Mercier et al. [75]. In brief, proteins were precipitated in two aliquots using 10% trichloroacetic acid (TCA, *w*/*v*) and centrifuged at 2000× *g* for 10 min. The resulting pellets were processed as follows: Aliquote 1 was treated with 1 mL of 2 M hydrochloric acid (HCl) as a control; Aliquote 2 was treated with 1 mL of 0.2% (*w*/*v*) 2,4-dinitrophenylhydrazine (DNPH). Both aliquots were incubated at room temperature for 1 h with regular stirring. Subsequently, they were re-precipitated with 10% TCA (*w*/*v*), centrifuged, and washed twice with 1 mL of ethanol/ethyl acetate (1:1) to remove residual DNPH and soluble lipids. The proteins were dissolved in 2 mL of 6 M guanidine hydrochloride, and any insoluble fragments were removed by centrifugation. Protein concentration was determined at 280 nm using bovine serum albumin (BSA, Sigma) as a standard, while carbonyl content was measured at 370 nm using a molar absorption coefficient of 22.0 L mmol^−1^ cm^−1^. Results were expressed as nmol of DNPH bound per milligram of protein.

### 5.9. SOD Activity Analysis

Fresh tissue samples (~100 mg) were rinsed with cold phosphate-buffered saline (PBS, pH 7.4) and homogenised on ice in 0.5 mL of cold buffer (20 mM HEPES buffer, pH 7.2, containing 1 mM EGTA, 210 mM mannitol, and 70 mM sucrose) using a Power Gen 125 Homogenizer (Fisher Scientific UK Ltd., Loughborough, UK). The homogenates were centrifuged at 1500× *g* for 5 min at 4 °C in an Eppendorf Centrifuge 5417 R. The supernatant fraction was separated and stored at −80 °C until further analysis. The total SOD activity was determined using the Superoxide Dismutase Assay Kit (Item No. 706002, Cayman Chemical Company, Ann Arbor, MI, USA) on a TECAN Infinite M2000 Pro microplate reader (TECAN, Grödig, Austria). Cayman’s Superoxide Dismutase Assay Kit utilises a tetrazolium salt for the detection of superoxide radicals generated by xanthine oxidase and hypoxanthine. SOD activity was expressed as units per milligram of protein in supernatants (U (mg protein)^−1^.

### 5.10. GPx Activity Analysis

Fresh tissue samples (~100 mg) were rinsed with a cold PBS solution, pH 7.4, and homogenised on ice in 0.5 mL of cold buffer (50 mM Tris-HCl, pH 7.5, 5 mM EDTA, and 1 mM DTT) using a Power Gen 125 Homogenizer (Fisher Scientific UK Ltd., Loughborough, UK). The homogenates were centrifuged at 10,000× *g* for 15 min at 4 °C in an Eppendorf Centrifuge 5417 R. The supernatant fraction was separated and stored at −80 °C until further analysis. The activity of GPx was determined using the Glutathione Peroxidase Assay Kit (Item No. 703102, Cayman Chemical Company, Ann Arbor, MI, USA) on a TECAN Infinite M2000 Pro microplate reader (TECAN, Grödig, Austria). Cayman’s GPx Assay Kit used Cumene hydroperoxide as a substrate. GPx activity was calculated based on the decrease in absorbance at 340 nm, corresponding to the oxidation of NADPH to NADP+ and expressed as U (mg protein)^−1^. Protein concentration in the supernatant was measured using the Bradford colourimetric assay [76] with bovine serum albumin BSA as the standard.

### 5.11. Statistical Analysis

Statistical analyses were performed using Statistica 10 (Dell, Round Rock, TX, USA). The normality of data distribution was assessed using the Kolmogorov−Smirnov and Shapiro−Wilk tests and by examining z-values for skewness and kurtosis. Data were considered normally distributed if the z-values for skewness and kurtosis fell within the range of ±1.96. For normally distributed data, a one-way ANOVA with Scheffé’s post hoc test was employed to evaluate differences in the studied parameters using a more conservative approach. For telomere length only, due to the experimental and methodological approach, we compared the control group with each treatment group only, using a two-way independent t-test. There were four animals (N = 4) per each experimental point. Statistical significance was set at *p* < 0.05. In all graphical representations, the results are expressed as the arithmetic mean ± standard deviation.

## Data Availability

Data available upon reasonable request.
